# Simultaneous Bilateral Neck of Femur Fracture After Spiritual Therapy

**DOI:** 10.7759/cureus.29469

**Published:** 2022-09-22

**Authors:** Mosa A Alzahrani, Mohammed Alsabieh, Hameed H Alzomor, Wael A Abdelrahman

**Affiliations:** 1 Orthopedic Surgery, Prince Mohammed Bin Abdulaziz Hospital, Riyadh, SAU; 2 Orthopedic Surgery, Mansoura University, Mansoura, EGY

**Keywords:** non-union, total hip athroplasty, malignment, electric shock, bilateral femoral neck fracture

## Abstract

Bilateral simultaneous fracture of the neck of the femur is an extremely rare injury; out of the reported cases, 50% are caused by electrical shock. We reported a rare case of simultaneous bilateral femur neck fracture caused by electrical shock as a part of spiritual therapy. The patient underwent bilateral open reduction and internal fixation with cannulated screws. Unfortunately, the fixation failed, and the patient underwent bilateral total hip arthroplasty. The patient was satisfied with the outcome at the final follow-up.

## Introduction

Neck of femur fractures usually require high mechanism injuries like road traffic accidents or falls from height in young healthy patients; on the other hand, neck of femur fractures in the elderly usually result from low mechanism injuries such as falls from standing height [1]. Simultaneous bilateral neck of femur fracture is a rare injury, particularly in non-pathological bone. This fracture occurs during the violent unopposed pull of the muscles in the tonic phase [2]. Several cases have been reported in the literature, with electrical shock and seizures being the shared etiology [3-10].

## Case presentation

**Figure 1:**
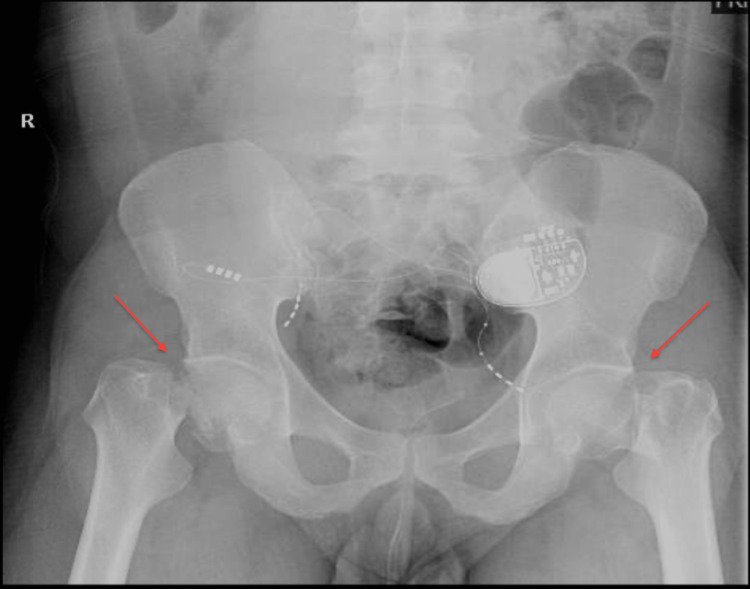
Bilateral neck of femur fracture x-ray

A 36-year-old male, smoker (20 pack years), was known to have urinary incontinence secondary to neurogenic bladder; the patient was otherwise healthy. The patient presented to our emergency department complaining of bilateral hip pain and inability to bear weight after he was shocked by an electrical current as a part of a spiritual therapy session. Before presenting to our hospital, the patient was bed-bound for one week and did not seek any medical attention. Upon presentation, the patient was fully awake, with tender hips; there was no open wound or burn. Bilateral legs were shortened and externally rotated with intact distal neurovascular status. X-rays showed bilateral neck of femur fracture (Figure 1).

Routine laps were obtained, and cardiology was involved to clear the patient for surgery as he had elevated cardiac enzymes (Table 1). The patient was taken for surgery one day after his presentation.

**Table 1:** Patient cardiac isoenzymes CK: creatine kinase

Cardiac isoenzymes	Result
CK-MB	153.0 U/L
CK	4267 U/L
Troponin-I	0.01 ng/mL

Surgical technique 

**Figure 2:**
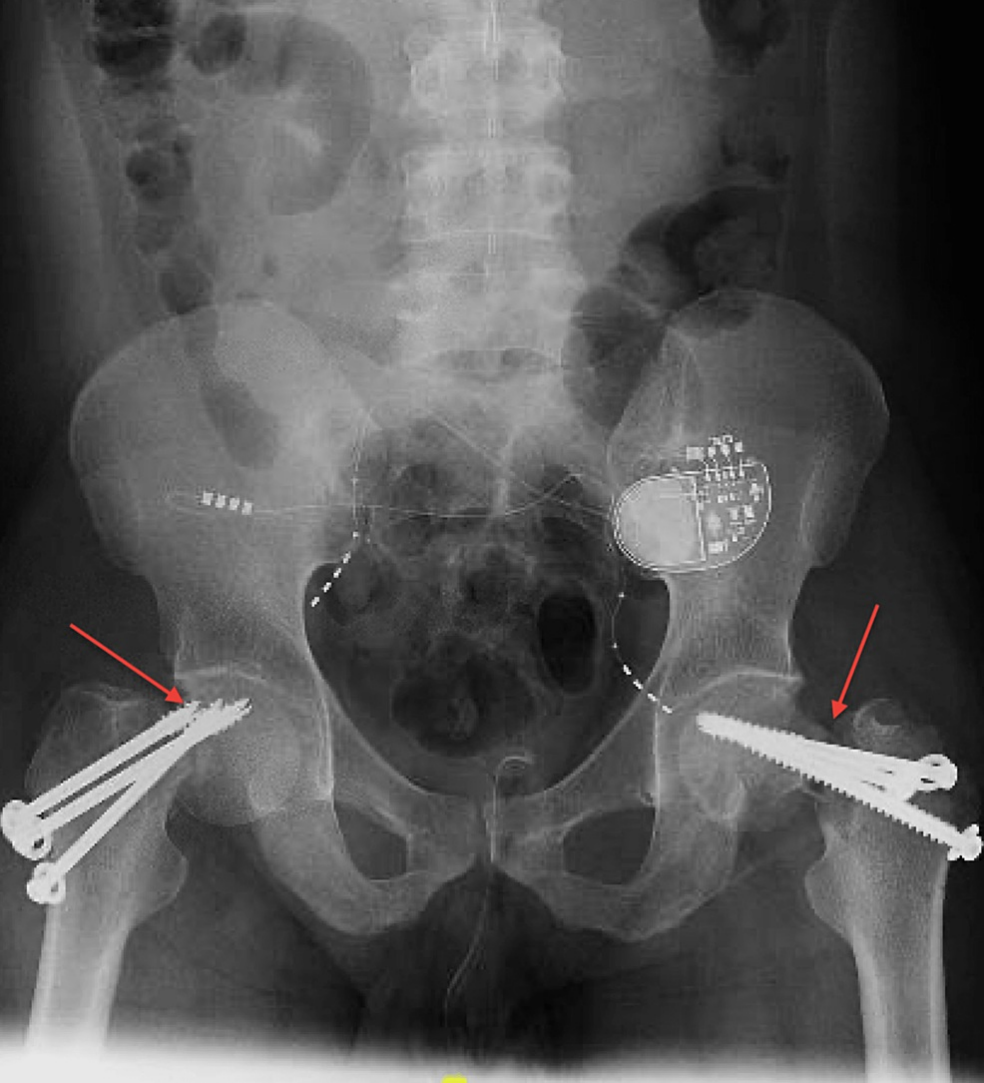
Postoperative x-ray after close reduction and cannulated screws

Under general anesthesia, the patient was put on a traction table. Leadbetter’s technique was attempted but proper reduction could not be achieved. Starting with the left side, prepping and draping were done in the usual sterile manner, 2 cm lateral thigh incision was utilized to advance three cannulated screws with washers, inserted under c-arm guidance, and irrigation of the wound was done with closure layer by layer. The same technique was used for the right side. The patient tolerated the surgery well and was shifted to the recovery room in stable condition. Postoperative x-rays showed malreduction fixation bilaterally (Figure 2).

**Figure 3:**
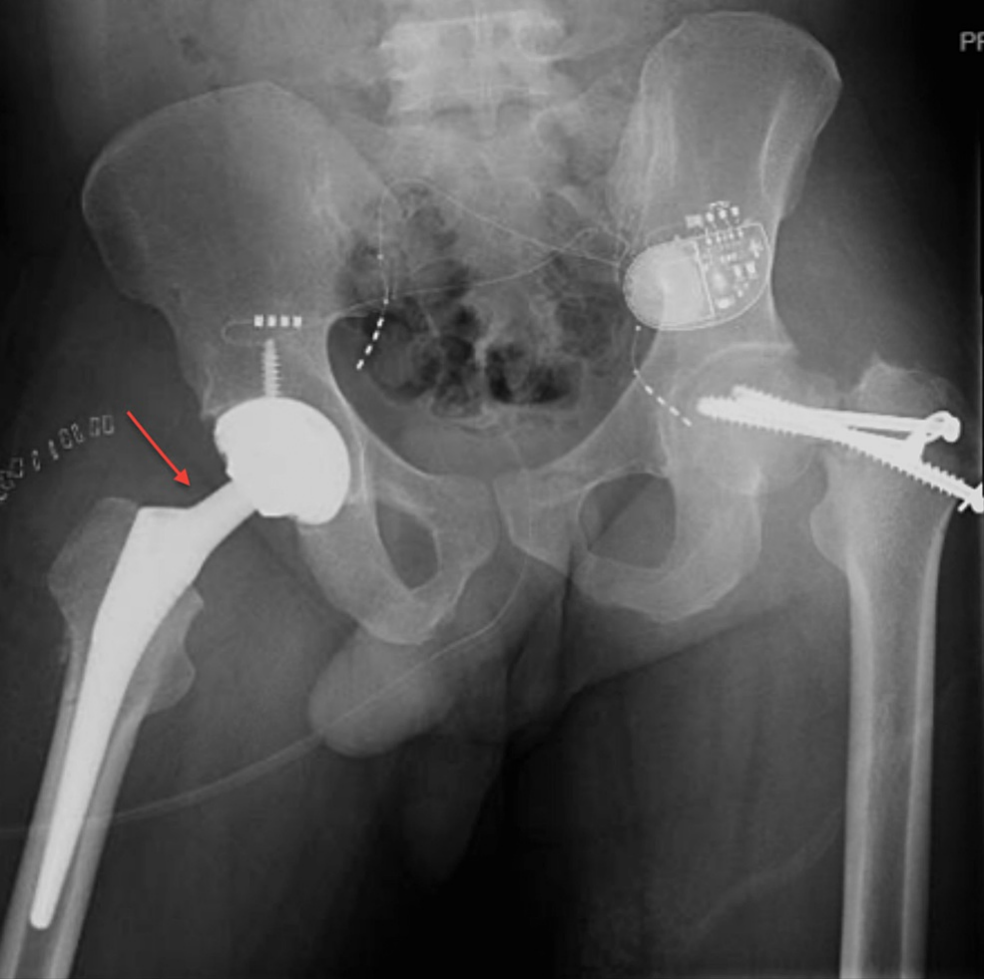
Postoperative right side total hip arthroplasty x-ray

**Figure 4:**
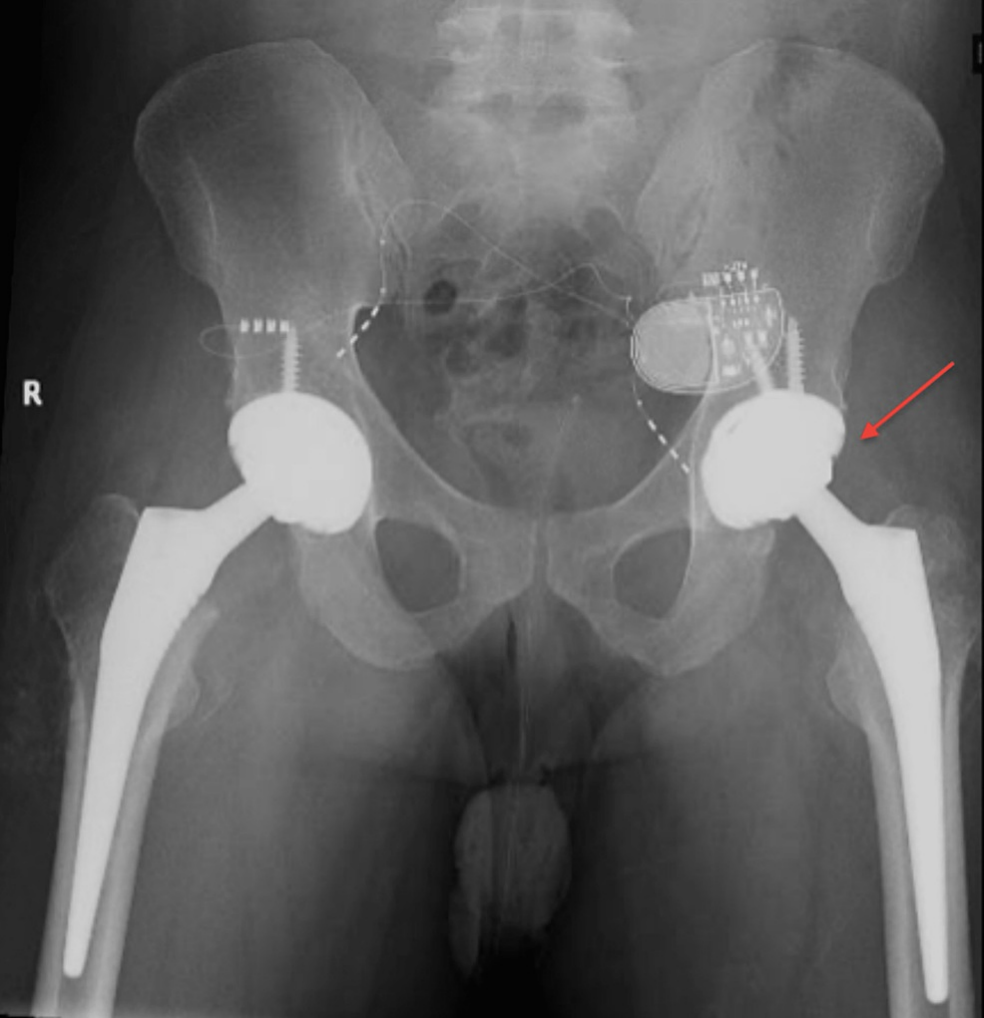
Postoperative left side total hip arthroplasty x-ray

**Figure 5:**
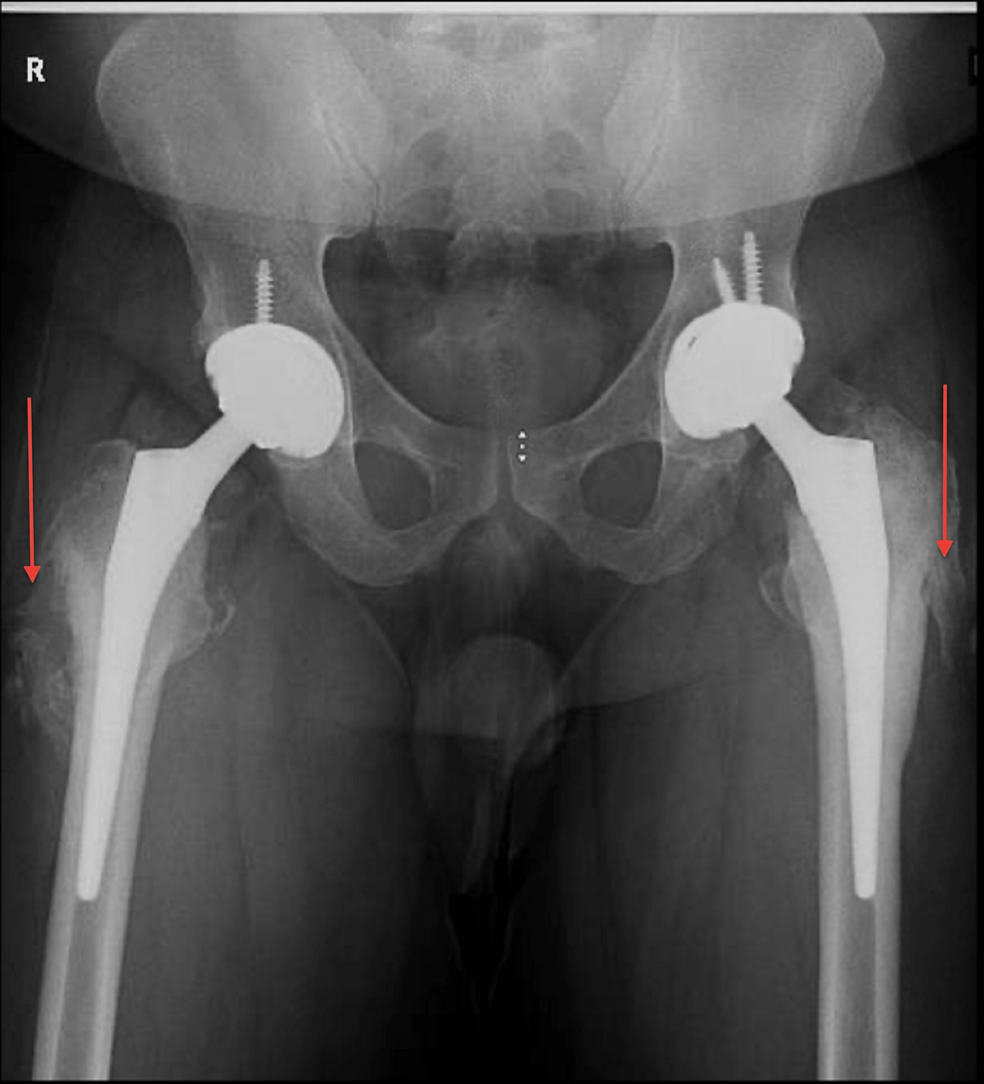
Follow-up x-ray after three years (arrows - heterotopic ossification)

Eleven days after the first procedure, the patient underwent bilateral total hip arthroplasty. The patient tolerated the surgeries well. He was instructed and educated to start full weight bearing with posterior hip precautions. Later, he was discharged on the third day of the surgery. He was followed up and seen regularly in our clinic, thereafter at his last follow-up (three years postoperatively), he was satisfied with the outcome including a full range of motion and no pain. The patient developed heterotopic ossification on plain radiographs but without clinical limitations. Immediate postoperative x-rays are shown in Figure 3 and Figure 4, and the x-ray at the final follow-up is shown in Figure 5.

## Discussion

Delay in the management of neck of femur fractures can negatively impact the quality of reduction and the final outcome. Late presentation, failure to investigate, distracting injuries, and difficulty in elucidating a clear history from a recently electrocuted patient are all possible justifications for the delay; this delay can range from days to weeks [11,12].

Electric shock can cause musculoskeletal injuries in different sites of the body as reported in the literature, for instance, posterior shoulder dislocation, fracture of proximal humerus, distal radius fracture, L4 burst fracture [12- 14]. Therefore, a careful examination from head to toe and proper imaging are crucial and have to be carried out to not miss those injuries. Our patient presented one week after the injury with no clear reason behind the delay; after further questioning, the patient admitted that he was electrically shocked as a part of exorcism treatment.

The neck of femur fractures has a tendency for non-union and it has to be surgically reduced well for a higher chance of union. To investigate the rates of non-union, Banks followed up a number of patients with displaced fractures who were treated by closed reduction and various types of internal fixation [15]. The incidence of non-union was 18% in 100 adequately reduced fractures and 70% in 23 inadequately reduced fractures. In our case, the patient initially underwent open reduction and internal fixation which resulted in mal-reduction that led to non-union. Later, he required total hip replacement to overcome and treat neck of femur non-union bilaterally. 

Total hip arthroplasty is superior to hemiarthroplasty in the treatment of femur neck fractures in regards to ambulation, pain, and repeated surgeries, as resulted by two large meta-analyses [16,17].

We reported a rare case of simultaneous bilateral neck of femur fracture that was caused by electrical shock. The fracture was malreduced and the patient underwent bilateral total hip arthroplasty on each side at a time. The patient was satisfied with the final outcome at the final follow-up of three years. The patient had multiple risk factors for heterotopic ossification (HO), including male gender, smoking, and neck of femur fracture [18]; he developed HO but fortunately, he was asymptomatic with nearly full range of motion.

## Conclusions

In rare circumstances, an electric shock may result in neck of femur fracture. Our case presented a simultaneous bilateral neck of femur fracture that was caused by electrical shock as a part of spiritual therapy. Open reduction and internal fixation failed initially and the patient underwent bilateral total hip arthroplasty. The patient was satisfied with the final outcome at the final follow-up. Failure of fixation was attributed to the late presentation hence early diagnosis and early fracture anatomical reduction and fixation are crucial to decrease potential complications such as avascular necrosis and fracture non-union.
